# Hybrid deep learning for smart paddy disease diagnosis using self supervised hierarchical reconstruction and attention based temporal analysis

**DOI:** 10.1038/s41598-025-18672-w

**Published:** 2025-10-07

**Authors:** Nagamani Thanuboddi, Usha Rani Nelakuditi

**Affiliations:** Vignan’s Foundation for Science, Technology and Research, Guntur, 522213 India

**Keywords:** Early disease detection, Self-supervised classification deep hierarchical reconstruction network (SSDHR), Long short-term memory (LSTM), Symmetric future attention (SFA), XGBooster, Engineering, Electrical and electronic engineering, Mathematics and computing, Computer science

## Abstract

Accurate and early disease detection in paddy crops is essential for maximizing crop yield which ensures food security. Traditional methods are often labor-intensive, time-consuming, and domain-specific expertise. Feed-forward deep-learning models will perform accurate disease detection through the identification of spatial patterns. However, they cannot predict the diseases at the early stages due to the lack of temporal information. Temporal observations will help perform continuous monitoring and detect minute changes in the crops at the early times. To tackle this problem, we proposed Self-Supervised Deep Hierarchical Reconstruction (SSDHR), and Long Short-Term Memory (LSTM) which perform early disease detection based on the spatial and temporal data respectively. The SSDHR network uses multi-branch convolution kernels to extract distinct discriminative characteristics rather than conventional leaf-based indicators. It incorporates spatial, and temporal-based attention mechanism Symmetric Fusion Attention (SFA) to improve feature selection and XGBoost (XGB) classifier for better stability. According to experimental findings, the suggested framework achieves a 99.25% accuracy rate in identifying and classifying 13 paddy classes, including normal, blast, hispa, tungro, white stem borer, brown spot, leaf roller, downy mildew, yellow stem borer, bacterial leaf blight, bacterial leaf streak, black stem borer, and bacterial panicle blight.

## Introduction

As a staple grain, paddy is essential to the world’s food security, particularly in nations like India, where it is a primary dietary component and a key economic driver. According to the International Rice Research Institute (IRRI), rice is the staple food for more than half of the world’s population and supports the livelihood of approximately 4 billion people globally^1,2^. Given the current per capita availability, paddy production needs to increase by 40–70%, from 116 to 145 and 190 million tons of milled paddy by 2030 and 2050 to meet growing demands Pradhan et al^3^. However, crop diseases pose a significant challenge to paddy production, affecting yield and quality. Crop diseases pose a significant challenge to paddy production, affecting both yield and quality. For example, a severe outbreak of paddy blast disease in Tamil Nadu in 2019 resulted in a yield loss of up to 30%, significantly disrupting local rice supply and causing economic hardship for farmers^4^. Early detection of such diseases is essential to reduce yield loss and ensure food security. Furthermore, incorporating both temporal and frequency domain analyses can provide deeper insights into disease progression. Major causes of crop loss such as plant diseases and pest infestations are known to be heavily influenced by environmental and agronomic factors^5,6^.

Poor water management creates conditions conducive to disease and pest proliferation^7^. Similarly, inadequate soil nutrition weakens plant health, making crops more vulnerable to infections^8^. Furthermore, climate variability leads to plant stress, increasing their susceptibility to biotic diseases and consequently reducing productivity^9^. The increased cultivation of high-yielding, fertilizer-responsive paddy varieties has further contributed to a rise in disease prevalence, especially during monsoon seasons^10^. Recent developments in deep learning (DL) and artificial intelligence (AI) have transformed the identification of plant diseases by providing automated, precise, and real-time disease diagnosis systems. Among these, deep learning models such as^11,12^ Convolutional Neural Networks (CNNs), Multi-Layer Perceptrons (MLPs), and Long Short-Term Memory (LSTM) networks have shown remarkable effectiveness in identifying diseases.

### Literature survey

A study on stress-tolerant breeding was conducted by Pradhan et al. in^3^. Strong performance has been shown by deep learning models like CNN and LSTM^13^ used a CNN-LSTM hybrid to identify apple illness with 92.3% accuracy, while Patil & Kumar^14^ introduced Paddy-Fusion, increasing accuracy to 94.7%. CNN-LSTM was used to improve illness severity categorization by Kukreja et al.^15^ and Kaur et al.^16^, who achieved 91.5% and 93.2% accuracy, respectively. Jiang et al.^17^ used image-based detection techniques that incorporated deep learning and SVM, achieving 90.1% accuracy. Kukreja. January^18^: presents a new model that blends Long Short-Term Memory (LSTM) networks with Convolutional Neural Networks (CNN). This model uses temporal and spatial data to accurately (94.06%) determine the severity of the condition. Dubey & Choubey^19,20^ used MBi-LSTM with adaptive feature selection, increasing classification accuracy to 95.4%. While Badgujar et al.^21^ enable AI-based categorisation, real-world datasets such as “PaddyDoctor,” created by Petchiammal et al. ^22^, The accuracy of the CNN-LSTM model is 94.5%, which is higher than that of the conventional CNN-based models, which are between 88 and 90% accurate. AI is beneficial to precision agriculture, as discussed in Agriculture 5.0 by Malekloo et al.^23^ and Mesías-Ruiz et al.^24^. The LSTM classifier and MLP-CNNs produced the greatest accuracy score of 99.2%. Choubey and Dubey^25^. Time-series forecasting for disease prediction, like CNN-LSTM-MLP models by Ghimire et al.^26^, achieved 92.8% accuracy, and Kontopoulou et al.^33^ examined ARIMA vs. ML models and found that ML outperformed conventional techniques. In their exploration of transfer learning using ResNet, VGG, and Inception, Shafiq & Gu^25^ and Pillai et al.^21^ achieved 96.1% accuracy in the categorization of plant diseases. According to Lu et al.^32^, hybrid models such as CNN-BiGRU enhanced the identification of paddy diseases with an accuracy of 94.2%, while Thapliyal et al.^40^ employed CNN-LSTM to detect maize diseases with an accuracy of 92.5%. To diagnose paddy nutrient levels during the panicle initiation stage, Liao et al.^24^ created a CNN-LSTM model, which outperformed conventional image-based techniques (88–91%) with an accuracy of 94.2%. Similar to this, Daniya & Vigneshwari^31^ demonstrated that hybrid feature extraction enhances illness identification by proposing a deep neural network that integrates texture-based (GLCM) and deep features, with 91.8% accuracy. In the meantime, Farooqui et al.^32^ improved illness classification accuracy to 93.5%^33^ by using a concatenated deep feature method with a modified LSTM, outperforming conventional CNN-LSTM models (89–91%).

Recent advancements in deep learning have significantly improved the accuracy and reliability of plant disease detection systems. Among these, proposed a multi-attention convolutional neural network (CNN) integrated with transfer learning techniques for the early classification of rice diseases. Their model leverages attention mechanisms at multiple stages of the CNN pipeline to focus on disease-relevant features in leaf images, improving both interpretability and precision. Furthermore, the use of transfer learning enabled the model to benefit from large-scale pre-trained weights, reducing the need for massive domain-specific data. Their results showed a substantial improvement in early detection accuracy, particularly under varied lighting and background conditions, making the model more applicable to real-world agricultural environments.

In another recent study, Zhou et al. ^34^ introduced a hybrid interpretable deep ensemble model designed specifically for rice disease identification under real field conditions. Their framework combines multiple deep learning models, including CNNs and attention-based networks, into a cohesive ensemble that enhances robustness and performance. A key contribution of this work is its emphasis on model interpretability, which is crucial in agriculture for gaining trust from end-users such as farmers and agronomists. The authors also validated their model on a complex dataset collected in natural field environments, which included challenges such as occlusion, noise, and varied lighting. The ensemble method outperformed individual models in terms of accuracy, stability, and resilience to noisy inputs, highlighting its practicality for deployment in smart farming systems.

While we acknowledge that many deep learning models have demonstrated high accuracy in plant disease classification, our choice of the Self-Supervised Deep Hierarchical Reconstruction (SSDHR) model integrated with LSTM and Symmetric Fusion Attention (SFA) was made deliberately based on the specific limitations of existing models and the unique requirements of early paddy disease detection.

Specifically, our decision is justified by the following reasons:

1. Limitation of existing models in temporal learning:
  Most existing feed-forward deep learning models primarily focus on extracting spatial features. Although they can identify disease-affected regions, they are less effective in detecting diseases at early stages because they lack temporal awareness. Our framework addresses this limitation by incorporating LSTM, which captures temporal dependencies and enables early disease prediction through continuous monitoring.
2. Self-supervised learning advantage
  We employ a Self-Supervised Deep Hierarchical Reconstruction (SSDHR) approach to learn robust representations from unlabelled data, which is particularly useful in agricultural settings where annotated datasets are scarce or expensive to obtain. This distinguishes our model from traditional supervised-only approaches.
3. Enhanced feature discrimination through SFA:
  The proposed Symmetric Fusion Attention (SFA) module fuses spatial and temporal attention to emphasize subtle but crucial disease features. This is a key differentiator from other models, which often rely solely on spatial cues and overlook inter-frame correlations.
4. High accuracy and stability with XGBoost:
  To further enhance the classification stability, we integrate XGBoost (XGB) as the final decision layer, which complements the learned deep features and provides improved generalization performance. Our framework achieves 99.25% accuracy across 13 distinct paddy classes, including multiple diseases and healthy samples, indicating strong performance.

In summary, while many models offer good accuracy, our model was chosen for its ability to bridge the spatial and temporal gap, perform early detection, and operate effectively with minimal supervision. These advantages make it better suited for real-world agricultural applications where early intervention is critical.

## Materials and methods

### Materials

**Fig. 1:**
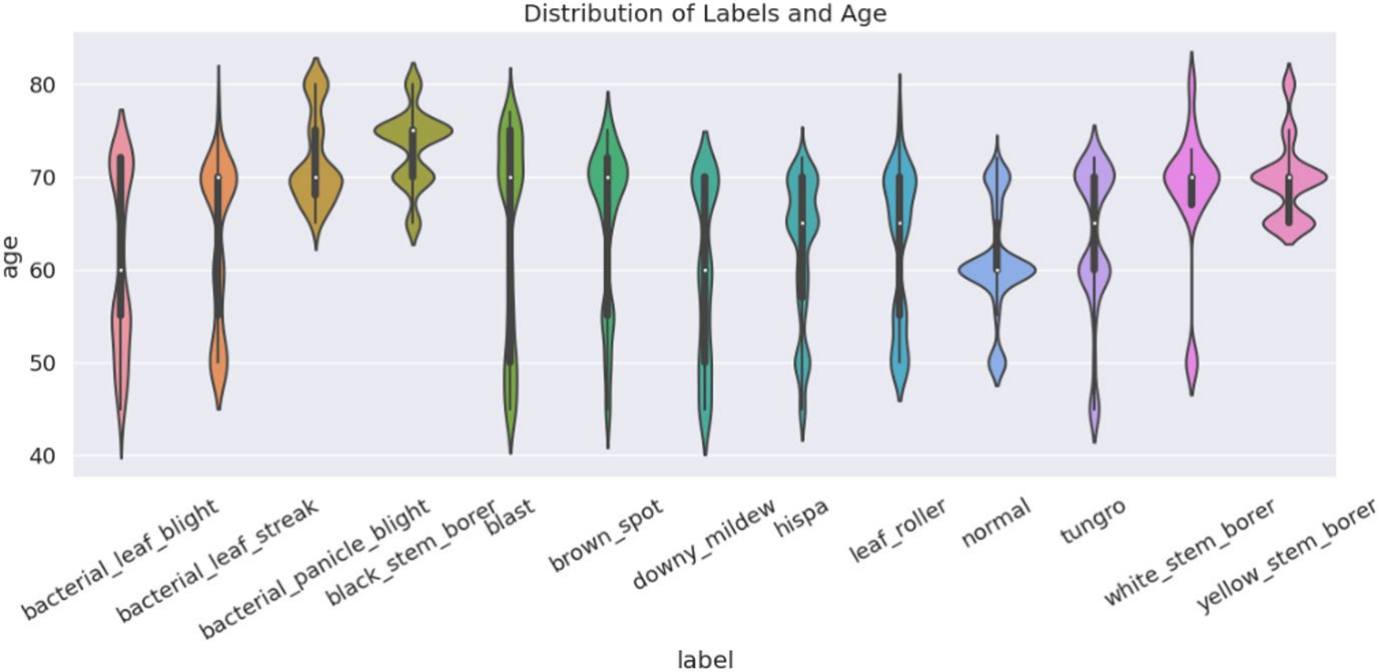
Paddy classes are distributed on days (age).

**Fig. 2:**
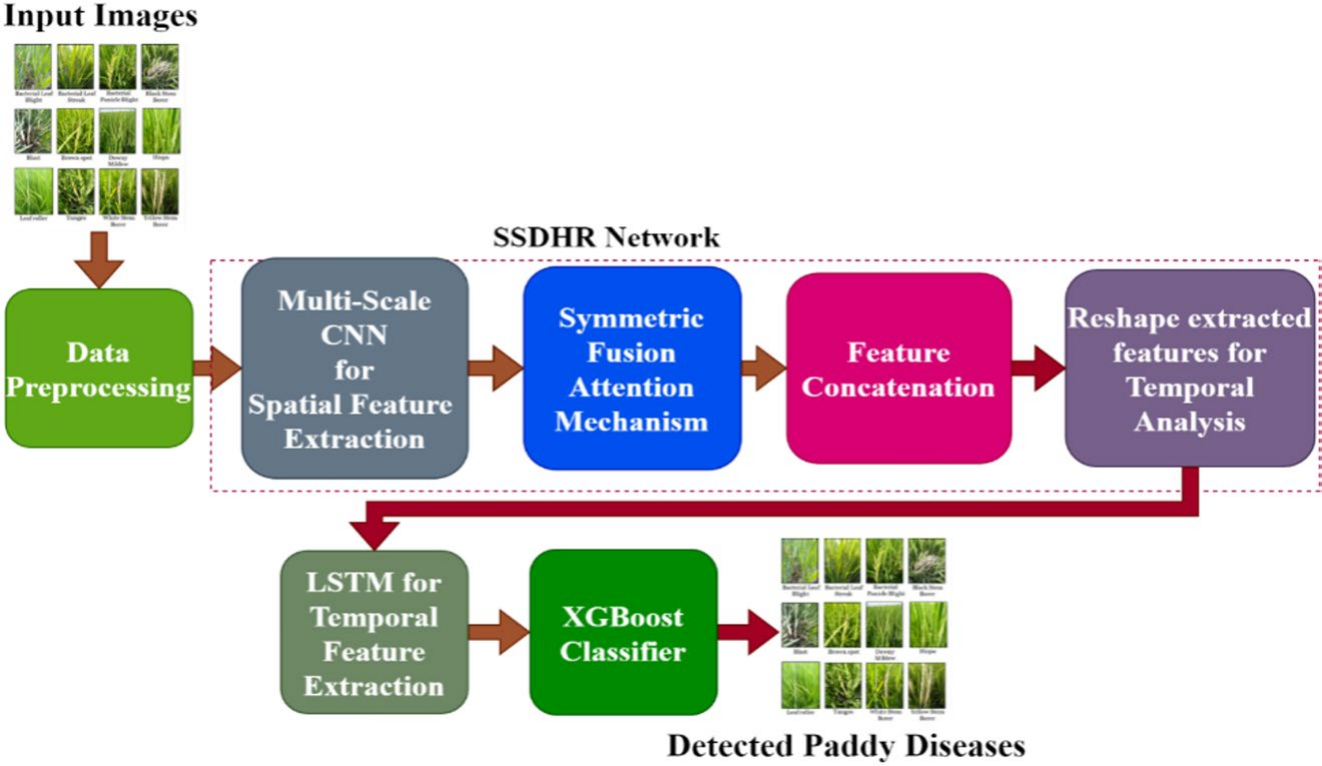
Working flow of the proposed model.

Paddy Doctor dataset data collected from IEEE Data port, following picture cleaning, our dataset consists of 16,225 images. Figure 2 depicts the data set, data collecting, and annotation procedure. Detailed information is shown in Table 1. In Fig. 1. violin plot displays the age distribution for each label. Labels are shown on the x-axis, while age is shown on the y-axis.

**Table 1:** Description of dataset.

Aspect	Details
Dataset name	IEEE data port
Source	Actual paddy fields near Tirunelveli district, Tamil Nadu, India
Timeframe	February–April 2021
Crop stage	Paddy crops aged 40–80 days
Final dataset size	16,225 high-quality images (after cleaning and filtering)
Verification	Expert agronomy specialists and plant pathologists
Training and testing	80% and 20%
Distribution	Balanced across disease categories and healthy samples
Lighting conditions	Natural lighting
Purpose	Paddy crop disease detection and benchmarking for machine learning and deep learning applications

The dataset used in our study was obtained from the publicly available repository titled “Paddy Disease and Pest Image Dataset” on IEEE Data Port. The dataset comprises 16,225 high-quality images across 13 classes, including 12 paddy disease and pest categories along with healthy samples. Below are the detailed aspects of the data collection methodology:

- Source & location: The images were collected from actual paddy fields near Tirunelveli district, Tamil Nadu, India, during the cropping season from February to April 2021.
- Crop stage: Paddy crops ranged in age from 40 to 80 days, covering the most disease-prone growth phases.
- Camera & resolution: Images were captured using the CAT S62 Pro smartphone, which supports both RGB and infrared imaging. The original resolution was 1080 × 1440 pixels, and the images were later resized to 480 × 640 pixels to optimize computational efficiency during training.
- Lighting conditions: All images were taken under natural lighting conditions, ensuring realistic field variability.
- Annotation process: The dataset was manually annotated by agricultural experts, including agronomy specialists and plant pathologists. Each leaf was categorized into one of the disease classes or as healthy.
- Initial cleaning: From an initial collection of over 30,000 images, noisy, blurred, and redundant samples were removed through manual inspection, resulting in the final curated set of 16,225 images.
- Metadata collection: Additional metadata such as paddy variety and crop age were also documented for each sample to enable more contextual analysis.

Dataset Link: https://ieee-dataport.org/documents/paddy-doctor-visual-image-dataset-automated-paddy-disease-classification-and-benchmarking.

The dataset comprises a total of 16,225 high-quality images, covering 13 categories (12 disease/pest classes and 1 healthy class). Each class contains 1248 images, ensuring minimal class imbalance. This distribution was carefully maintained during dataset creation to support fair model training and evaluation is shown in Table 2.

**Table 2:** Distribution of each class for model training and evaluation.

Class	Total	Train (66.4%)	Test (33.6%)
Blast	1,248	828	420
Hispa	1,248	828	420
Tungro	1,249	829	420
White stem borer	1,250	830	420
Brown spot	1,250	830	420
Leaf roller	1,248	828	420
Downy mildew	1,248	828	420
Yellow stem borer	1,250	830	420
Bacterial leaf blight	1,248	828	420
Black stem borer	1,250	830	420
Bacterial leaf streak	1,248	828	420
Bacterial panicle blight	1,248	828	420
Healthy	1,250	830	420
Total	16,225	10,777	5,448

A violin plot representation of the distribution of plant ages across 13 different paddy crop classes, including both healthy (normal) and diseased categories such as blast, hispa, tungro, bacterial leaf blight, brown spot, among others is shown in Fig. 1. The x-axis denotes the class labels, while the y-axis shows the crop age (in days), ranging from approximately 40 to 85 days. The shape and width of each violin illustrate the density of samples at specific age intervals, with wider sections indicating higher sample concentrations. Median values are shown as white dots, and interquartile ranges (IQR) are marked by thick black bars, providing insights into the central tendency and variability of each class. Notably, classes such as blast, bacterial panicle blight, and black stem borer exhibit relatively narrow age distributions, indicating that these diseases predominantly affect crops at specific stages. In contrast, the normal and leaf roller classes demonstrate broader age variability, suggesting their occurrence across a wider growth range. Moreover, the multimodal distributions observed in classes like tungro and yellow stem borer suggest disease incidence across multiple crop stages. This figure underscores the age dependency of various paddy diseases and highlights the need for incorporating temporal information in the model.

### Methods

The proposed method integrates a Self-Supervised Deep Hierarchical Reconstruction (SSDHR) network, a Symmetric Fusion Attention (SFA) module, and a Long Short-Term Memory (LSTM) network to generate robust features before classification using XGBoost. Accurate detection of paddy diseases requires extracting spatial features by SSDHR and temporal features by LSTM from images and time-series data and the SFA module refines the features. The details of the network are shown in Fig. 2.

To improve quality, data pre-processing (resizing, normalisation, augmentation) comes first is used. A symmetric fusion attention approach is used to decrease noise and retain pertinent information after a multi-scale CNN extracts spatial features. Temporal illness development is captured by concatenating and reshaping features for LSTM processing. Accuracy is increased by utilising XGBoost to classify the refined features. The model categorises paddy illnesses, such as Healthy, Tungro Virus, Brown Spot, Blast, and Bacterial Blight. This comprehensive strategy guarantees precise, instantaneous illness identification.

### Self-supervised classification deep hierarchical reconstruction network with symmetric fusion attention

The planned SSDHR classifier network structure. To provide a larger feature pool for plant disease classification, the authors of^35^ simultaneously used classification features y and the reconstructed latent representation z for closed-set classification as well as identification. Latent representations are extracted from every step of the middle-level layers in the classification network in the article, as a result of the loss of data details in the higher-level layers of supervised deep networks.

**Fig. 3:**
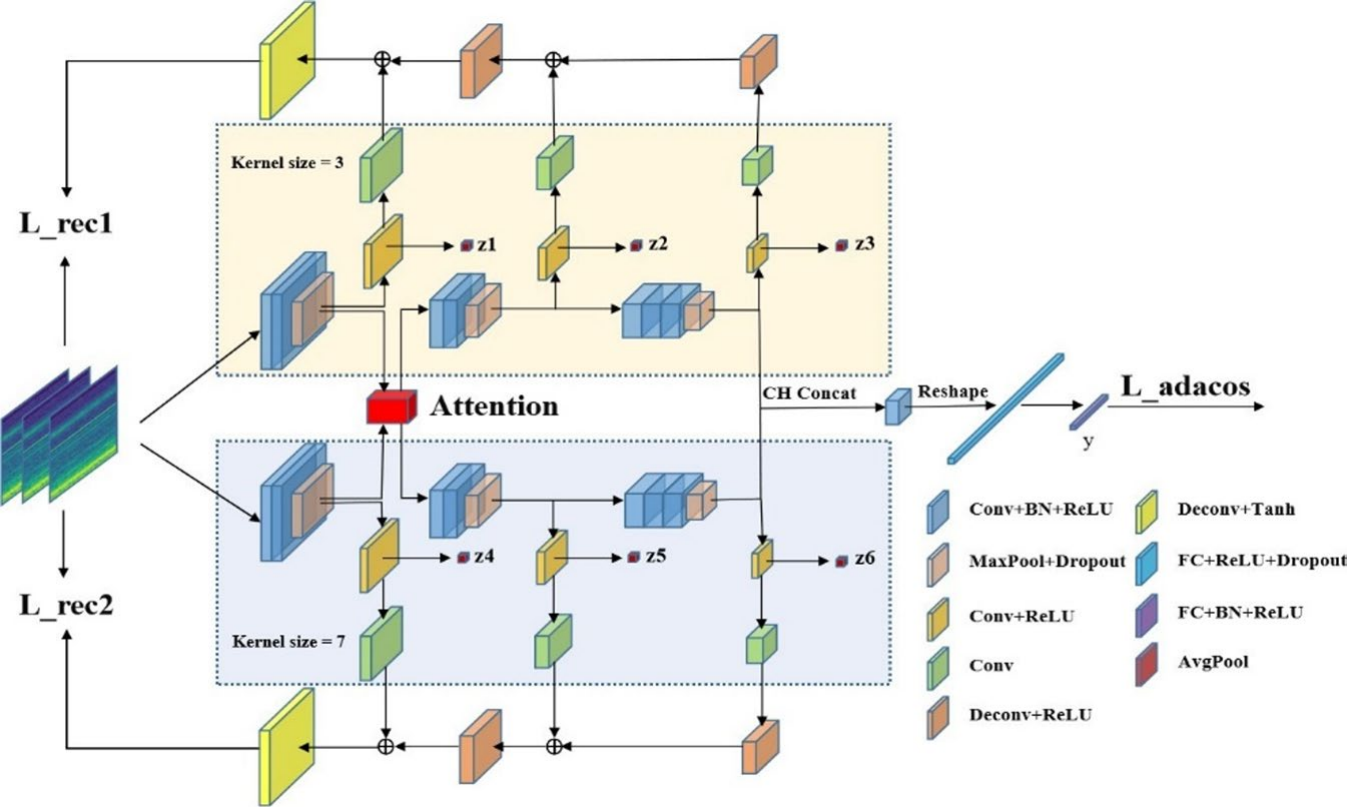
The architecture of SSDHR network.

In Fig. 3., two symmetric branches have convolution kernels of different sizes (3 × 3 and 7 × 7). The features “z1, z2, z3, …, z6” represent the pooled results of multiple reconstructed latent representations. “Attention” represents the proposed SFA module. The feature “y” represents classification features.

The model’s resilience and perception are improved by adding two parallel branches that use convolutional kernels of varying sizes for multi-scale feature extraction. The channel dimension is used to concatenate characteristics from both branches and recreate intermediate layers for richer representations. These are categorized by processing them through completely connected layers. A Symmetric Fusion Attention (SFA) module enhances performance by improving focus on important aspects. Mixed Centre Loss also improves accuracy by refining features for more biased and condensed representations.

### Symmetric fusion attention

Symmetric Fusion Attention (SFA) is a custom-designed attention mechanism developed to enhance the model’s ability to capture discriminative features from paddy leaf images by integrating both low-level and high-level spatial information. The “symmetric” nature refers to the balanced fusion of features extracted from multiple layers of the network (e.g., early and late convolutional layers), ensuring that fine-grained texture and contextual patterns are preserved. This attention module selectively emphasizes disease-relevant regions in the image, improving classification performance, especially in visually complex or overlapping disease cases.

**Fig. 4:**
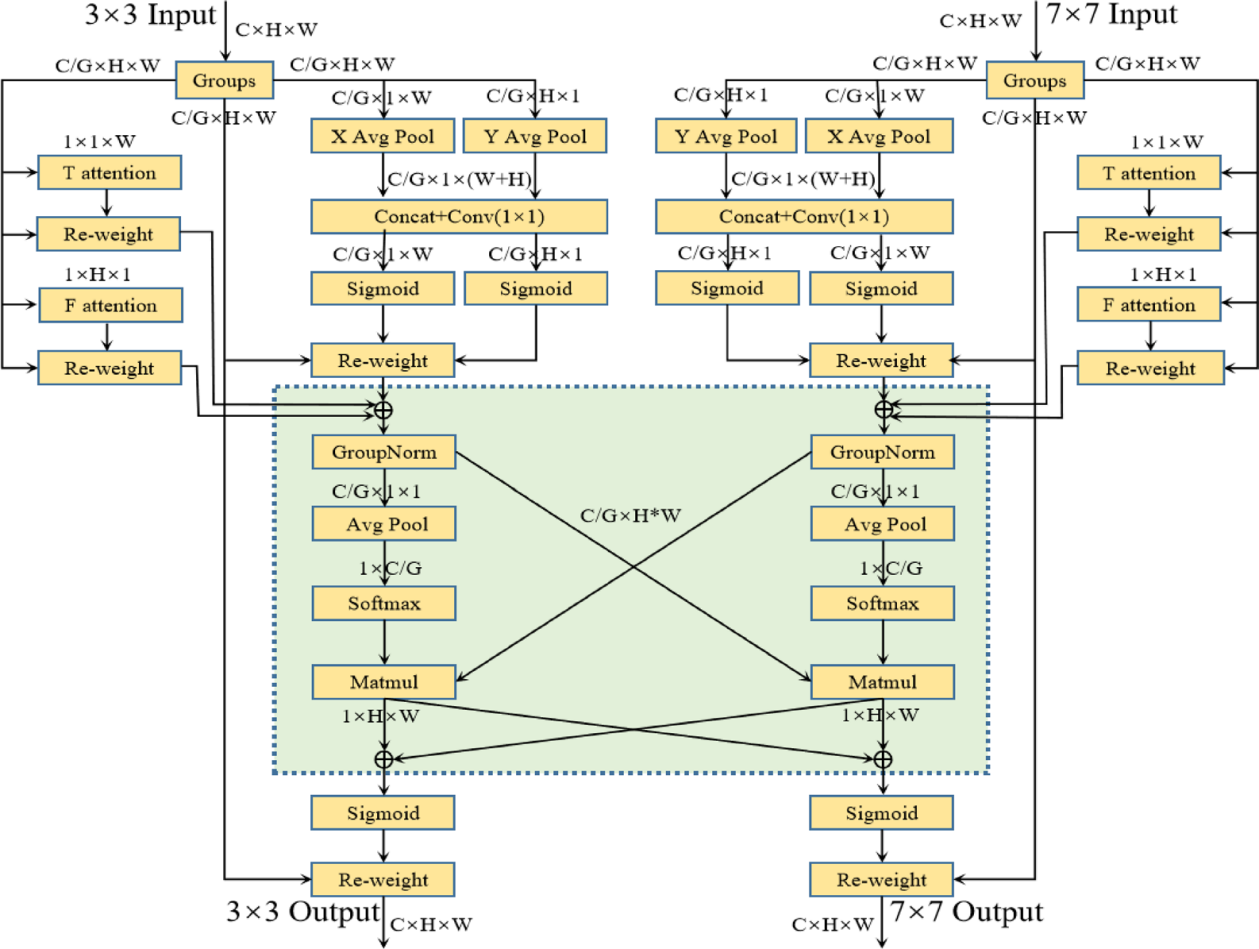
SFA model architecture.

The general layout of the attention module, which is suitable for parallel structured networks is shown in Fig. 4. To preserve information throughout each channel and lower computational overhead, restructure a portion of the channels for each input feature X $\in {\mathbb{R}}$ ^CxHxW^ into batch dimensions^36,37^. Instead of adding more branching structures, use the network’s initial parallel branches to capture multi-scale information. Additionally, each branch attention to both spatial and temporal aspects.

Figure 4 shows how these two attentions are computed. As a result, temporal attention,

“G” stands for the split groupings. One-dimensional vertical global pooling and one-dimensional horizontal global pooling are denoted by “X Avg Pool” and “Y Avg Pool,” respectively. Temporal attention is represented by “T attention” and frequency attention by “F attention.” The terms “3 × 3 Input” and “7 × 7 Input” denote inputs with various scales frequency attention, and coordinate attention are the three sub-branches that each branch of the symmetric structure simultaneously includes. Coordinate attention is a variation on CA attention^38,39^, in which inputs are processed via one-dimensional vertical and horizontal global pooling rather than global average pooling. This allows for the preservation of exact positional information along one spatial guidance while collecting long-range dependencies along another. At height h, the c-th channel’s output can be shown as:

1 $${\text{Uhc }}\left( {\text{h}} \right) = \frac{1}{W}\mathop \sum \limits_{0 \le J \le H} y_{{\text{c}}} { }\left( {{\text{h}},{\text{i}}} \right)$$

The output of the c-th channel at width w can be represented as:

2 $${\text{Uwc }}\left( {\text{w}} \right) = \frac{1}{H}\mathop \sum \limits_{0 \le J \le H} y{ }_{{\text{c}}} \left( {{\text{j}},{\text{w}}} \right)$$

where yc indicates the input features at the c-th channel.

We perform 1 × 1 convolutional processes and a sigmoid activation function after pooling the reshaped input features in two directions. This produces two sets of coordinate attention weights $P_{a}^{h} \in {\mathbb{R}}^{{\frac{C}{G} \times H \times 1}}$ and $P_{a}^{w} \in {\mathbb{R}}^{{\frac{C}{G} \times w \times 1}}$ in distinct directions. Coordinate attention can be thought of as independently integrating time and frequency information into the channel dimension when the model’s input is spectrograms. Furthermore, the following are the formulas for the previously introduced frequency attention Fa $\in {\mathbb{R}}^{1\times H\times 1}$ and temporal attention is Ta $\in {\mathbb{R}}^{1\times w\times 1}$ :

3 $${\text{T}}_{{\text{a}}} = \, \left( {\sigma \left( {{\text{AvgPool}}_{{({1},{1})}} \left( {{\text{Conv2D}}_{{{\text{1x1}}}} \left( {{\text{X}}_{{\text{G}}} } \right)} \right)^{{{\text{T1}},{3}}} } \right)} \right)^{{{\text{T1}},{3}}}$$

4 $${\text{F}}_{{\text{a}}} = \, \left( {\sigma \left( {{\text{AvgPool}}_{{({1},{1})}} \left( {{\text{Conv2D}}_{{{\text{1x1}}}} \left( {{\text{X}}_{{\text{G}}} } \right)} \right)^{{{\text{T1}},{2}}} } \right)} \right)^{{{\text{T1}},{2}}}$$

where AvgPool (1,1) represents adaptive average pooling, which reduces each channel’s feature map to 1 × 1, (σ) represents the sigmoid activation function, (.)T1,3 and (.)T1,2 represents transpose operations on dimensions 1 and 3, and dimensions 1 and 2, respectively. XG $\in {\mathbb{R}}^{{\frac{C}{G} \times w \times H}}$ represents the reshaped input feature map.

**Fig. 5:**
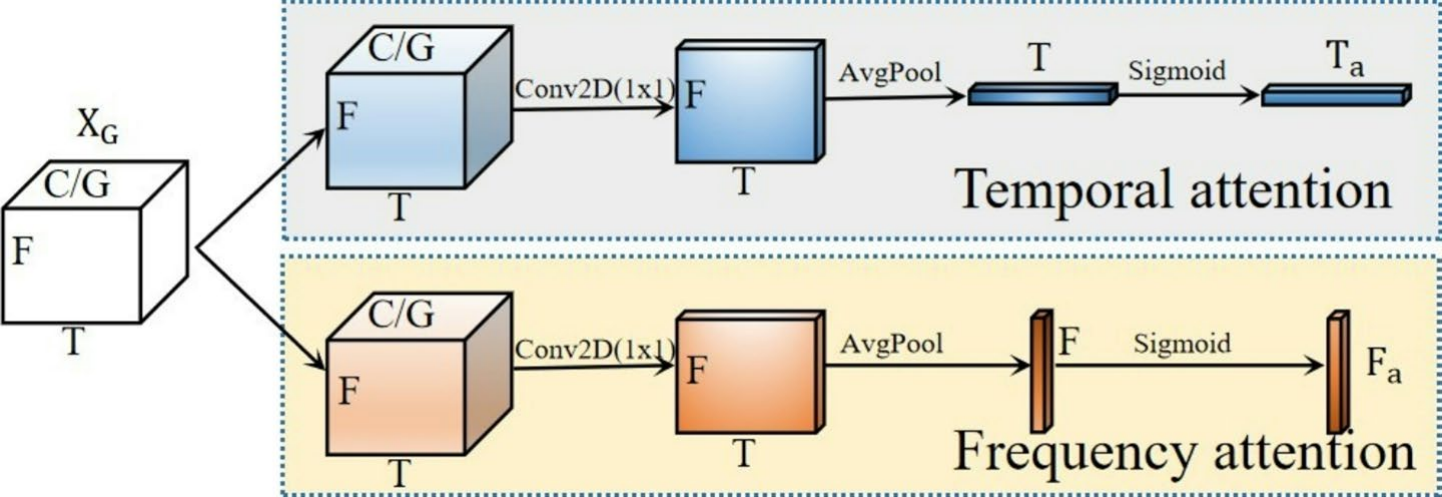
Temporal attention and frequency/spatial attention.

By applying temporal attention, frequency attention, and coordinate attention to XG and then weighting them, the output X’G $\in {\mathbb{R}}^{{\frac{C}{G} \times w \times H}}$ is obtained:

5 $$X^{\prime}G = \alpha X_{G} p_{a}^{h} p_{a}^{\omega } + \beta X_{G} Ta + \gamma X_{G} Fa$$

6 $$\alpha +\beta +\gamma =1$$

where $\alpha ,\beta ,\gamma$ are learnable parameters, and softmax function is applied to normalize them. Following that, information aggregation across several spatial dimension orientations is done cross-spatially. To achieve outputs, the two symmetrical branches employ temporal, frequency, and coordination attention simultaneously as shown in Fig. 5. The outputs of the two parallel branches are then subjected to two-dimensional global average pooling, and the pooled outputs are then reshaped to form ${\mathbb{R}}^{{1x\frac{C}{G}}}$. The outputs of both branches are simultaneously reshaped to dimensions ${\mathbb{R}}^{{\frac{C}{G}_{xHW} }}$ ^21^. To obtain two spatial attention maps on each branch, respectively, global average pooling is applied to the outputs, then softmax is applied and cross-branch matrix dot product operations are performed. The final shared spatial attention weights for the two branches are calculated by adding the two spatial attention maps and using the sigmoid function. To create outputs that are the same size as the inputs for both branches, these weights are multiplied by the inputs of the two branches.

### LSTM

LSTM networks allow for long-term dependency learning by processing sequential data by keeping a memory cell under the control of input, forget, and output gates. They work well for language translation, speech recognition, and time series forecasting. They can also be used in conjunction with CNNs to analyze images and videos.

**Fig. 6:**
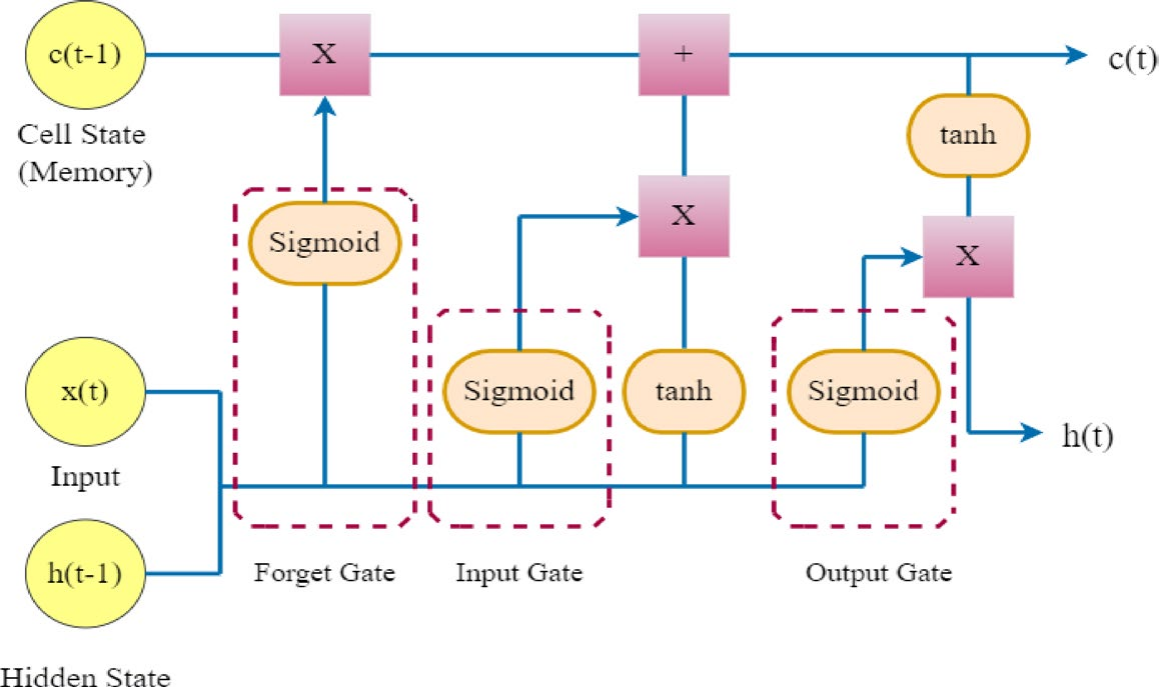
Architecture of LSTM.

In Fig. 6 shows Three gates provide control over the memory cell: the input gate, forget gate, and output gate. These gates determine which data should be input into, taken out of, and output from the memory cell. Information added to the memory cell is regulated by the input gate. The data that is deleted from the memory cell is managed by the forget gate. Additionally, what data is output from the memory cell is controlled by the output gate. In order to learn long-term dependencies, LSTM networks are able to selectively preserve or reject information as it moves through the network.

### XGBoost classifier

After feature extraction, the final feature vector F is used as input for XGBoost (eXtreme Gradient Boosting), an optimized gradient boosting algorithm that constructs an ensemble of decision trees. XGBoost predicts the disease class yi’ for a given input xi by aggregating multiple decision trees:

7 $$y_{{\hat{i}}} = \sum\limits_{k = 1}^{K} {f_{k} } \left( {x_{i} } \right)$$

where, K is the number of trees, ${f}_{k}\left({x}_{i}\right)$ represents the k-th decision tree, and $y_{{\hat{i}}}$ is the predicted disease label. Each tree is trained iteratively to minimize the objective function:

8 $$L = \sum\limits_{i = 1}^{n} l \left( {y_{i} ,y_{{\hat{i}}} } \right) + \sum\limits_{k = 1}^{K} \Omega \left( {f_{k} } \right)$$

where, $l\left( {y_{i} ,y_{{\hat{i}}} } \right)$ is the loss function and $\Omega \left({f}_{k}\right)$ is the regularization term controlling tree complexity. The gradient boosting update rule is:

9 $${f}_{k}=argmin \sum_{i=1}^{n}l\left({{y}_{i},y}_{i}^{^(k-1)}\right)+f\left({x}_{i}\right)+\Omega \left(f\right)$$

By integrating SSDHR + SFA + LSTM for feature extraction with XGBoost for classification, the proposed method achieves state-of-the-art accuracy in detecting 13 paddy types. This hybrid approach enhances model generalizability, making it suitable for real-time crop health monitoring and early disease detection.

### Loss function

In this section, we present the total loss function for the proposed LSTM-integrated SSDHR model for disease detection. The total loss function obtained by combining center loss, reconstruction loss, and mixed center loss of LSTM with the classification loss (AdaCos), of SSDHR model.

#### One-class Centre loss

The Centre Loss aims to increase the discriminative power of features by increasing the distance between feature points of different classes and decreasing the distance between feature points of the same class in the feature space. This is done by minimizing the separation between each sample and its corresponding class center. For disease recognition in paddy crops, this loss helps the model focus on learning robust disease features, improving classification accuracy.

10 $${L}_{ctr}=\frac{1}{2}{\sum_{j=1}^{n}\| {f}_{j}-{\mu }_{yj }\| }^{2}$$

where, fj ∈ Rd is the jth feature vector belonging to the yjth class. μyj ∈ Rd is the center of deep features for the yjth class. d is the feature dimension. This loss ensures that the deep features of the samples are clustered around the class centers, improving class separation.

#### One-class Centre loss (for normal samples)

The One-Class Centre Loss computes the distance between each feature and a global feature center for all normal samples (non-disease cases).

11 $${L}_{octr}=\frac{1}{2}{\sum_{j=1}^{n}\| {f}_{j}-{\mu }_{ }\| }^{2}$$

where, μ is the global feature center for all normal samples.

#### Mixed Centre loss

The Mixed Centre Loss combines both the center loss for the disease class and the one-class center loss for normal samples, giving us the final mixed loss.

12 $${L}_{mixctr}= {\uplambda }_{1}{L}_{ctr}+{\uplambda }_{2}{L}_{octr}$$

where, λ_1_,λ_2_ are scalar weights to balance the influence of both losses.

#### Reconstruction loss and classification loss

The network integrates reconstruction loss (based on Mean Squared Error) and classification loss (using AdaCos loss). This combination ensures that the network can both reconstruct the input features and classify them correctly.

### Reconstruction loss (MSE)

The network has two parallel branches for reconstructing the latent representations of the input data. The Mean Squared Error (MSE) for each branch is computed as follows:

For the first branch:

13 $$L_{rec1} = \frac{1 }{n}\mathop \sum \limits_{j = 1}^{m} \left\| {A_{j} - A^{\prime}_{j} } \right\|^{2}$$

For the second branch:

14 $$L_{rec2} = \frac{1 }{n}\mathop \sum \limits_{j = 1}^{m} \left\| {A_{j} - A^{\prime\prime}_{j} } \right\|^{2}$$

where, A_j_ is the input feature (such as a spectrogram of disease features), Aj′ and ‘‘Aj′′ are the reconstructed features from the first and second branches, respectively.

#### AdaCos loss (classification loss)

AdaCos Loss ensures that the model maintains a margin between different classes. This helps in reducing intra-class variance and maximizing inter-class variance. The probability Pi,j that sample Aj belongs to class j is computed using the following formula:

15 $${P}_{i,j}=\frac{{e}^{\widetilde{S}.cos\theta i,j }}{\sum_{k=1}^{k}{e}^{\widetilde{s}.cos\theta i,j}}$$

where, cosθi,j is the cosine similarity between input feature Xi and the class center wj, S ~ is a scaling factor.

The dynamically adaptive scaling parameter at step t is computed as:

16 $${\widetilde{S}}_{d}^{\left(t\right)}=\left\{\begin{array}{c}\sqrt{2}\mathit{log}\left(k-1\right)\\ \frac{\mathit{log}{B}_{{a}^{v}y}^{\left(t\right)}}{\mathit{cos}\left(\mathit{arccos}\left(\frac{\pi }{4},\theta q\right)\right)}\end{array}\right.$$

17 $${B}_{a}{v}_{g}^{(t)}=\frac{1}{N}\sum_{\text{i}\in {N}^{\left(l\right)}}\sum_{k\ne {y}_{i}}{\text{e}}^{{s}_{d}^{-l}t-1},\text{cos}{o}_{i,k}$$

#### Total loss function

The final total loss function Ltotal for the SSDHR model, combining all components, is given by:

18 $${L}_{total}={L}_{rec1}+{L}_{rec2}+{L}_{AdaCos}+{L}_{mixctr}$$

This comprehensive loss function ensures that the model performs both feature learning and disease classification effectively while accounting for the temporal nature of disease progression using the LSTM architecture.

## Results and discussion

Implemented proposed SSDHR integrated with LSTM architecture was implemented and validated with the Paddy data set. Including temporal and spatial attention mechanisms enhances the feature selection and improve the metrics such as accuracy, precision, recall, and F1-score are analyzed, and the results are compared with baseline deep learning models to demonstrate the effectiveness of our approach.

**Fig. 7:**
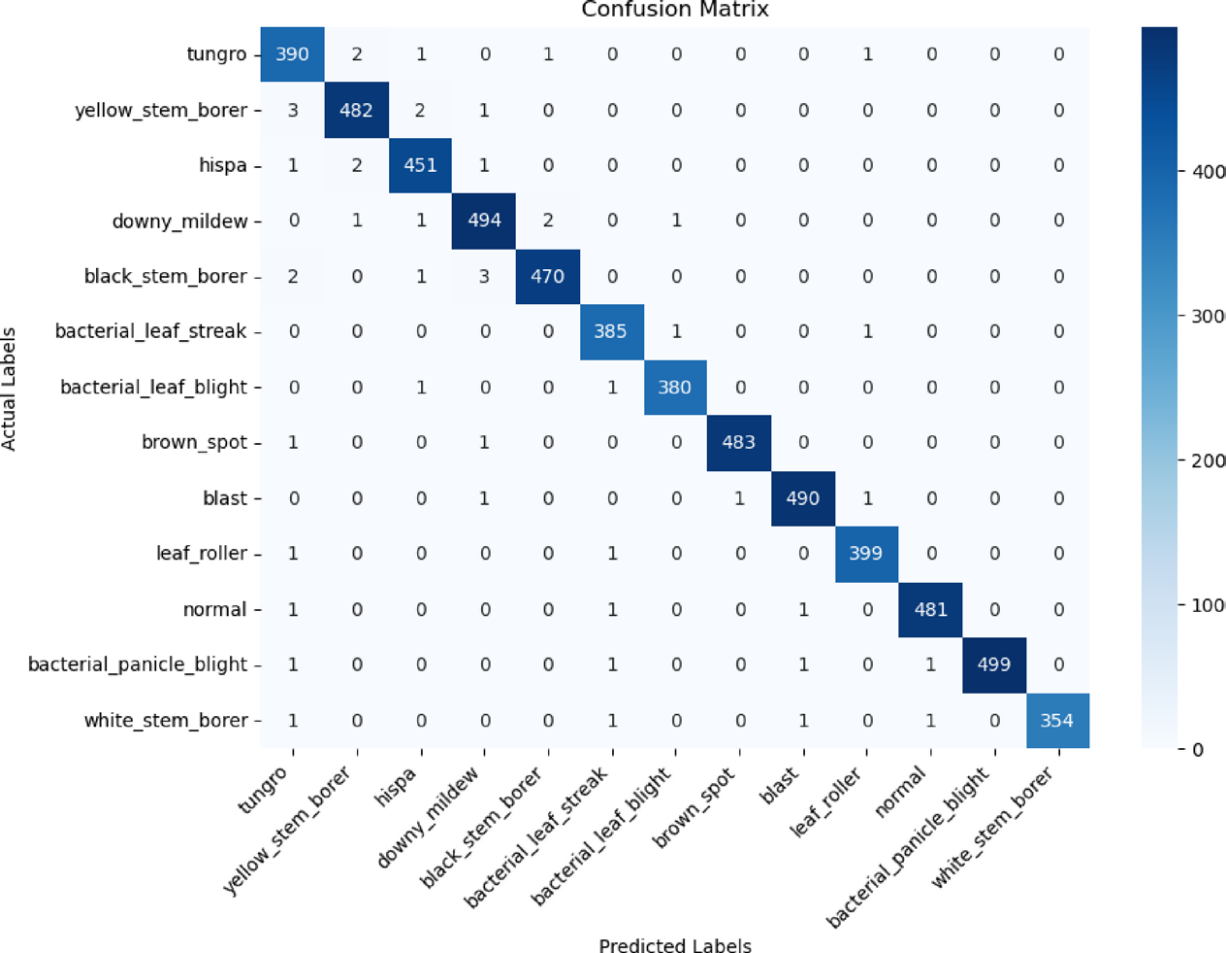
Performance comparison matrix between predicted and actual paddy disease classes.

Figure 7 is a confusion matrix, a widely used tool for evaluating the performance of classification models. The classification performance parameters, such as precision, recall, and F1-score, are shown in the table for both healthy samples and different crop disease groups. The precision values, which range from 0.9841 to 0.9993, demonstrate how well the model can identify each condition without producing an overwhelming number of false positives.

These values were obtained over five independent runs using fivefold cross-validation, which ensures a robust assessment of the model’s performance variability across different data splits is shown in Table 3.

**Table 3:** Proposed model’s performance of each class across different data splits.

Class	Precision (mean ± std)	Recall (mean ± std)	F1-score (mean ± std)
Blast	0.995 ± 0.002	0.993 ± 0.004	0.994 ± 0.003
Hispa	0.997 ± 0.005	0.993 ± 0.002	0.995 ± 0.004
Tungro	1.000 ± 0.004	0.992 ± 0.002	0.996 ± 0.002
White_stem_borer	0.992 ± 0.003	0.995 ± 0.002	0.993 ± 0.003
Brown_spot	0.975 ± 0.002	0.987 ± 0.002	0.981 ± 0.003
Lear_roller	0.993 ± 0.002	0.987 ± 0.003	0.990 ± 0.001
Downy_mildew	0.994 ± 0.001	0.994 ± 0.003	0.994 ± 0.003
Yellow_stem_borer	0.989 ± 0.004	0.987 ± 0.002	0.988 ± 0.002
Bacterial_leaf_blight	0.989 ± 0.003	0.994 ± 0.003	0.992 ± 0.001
Black_stem_borer	0.986 ± 0.004	0.991 ± 0.002	0.989 ± 0.005
Bacterial_leaf_streak	0.986 ± 0.001	0.989 ± 0.002	0.988 ± 0.005
Bacterial_panicle_blight	0.997 ± 0.005	0.991 ± 0.002	0.991 ± 0.004

The model has a great ability to capture true positives, as seen by the continuously high recall values (which assess its ability to properly recognize all instances of an illness), with a minimum of 0.9881. Additionally, the F1 scores, which strike a compromise between recall and precision, are above 0.9870, indicating strong classification performance in every class. The model’s total accuracy of 0.9953 shows remarkable classification reliability.

**Fig. 8:**
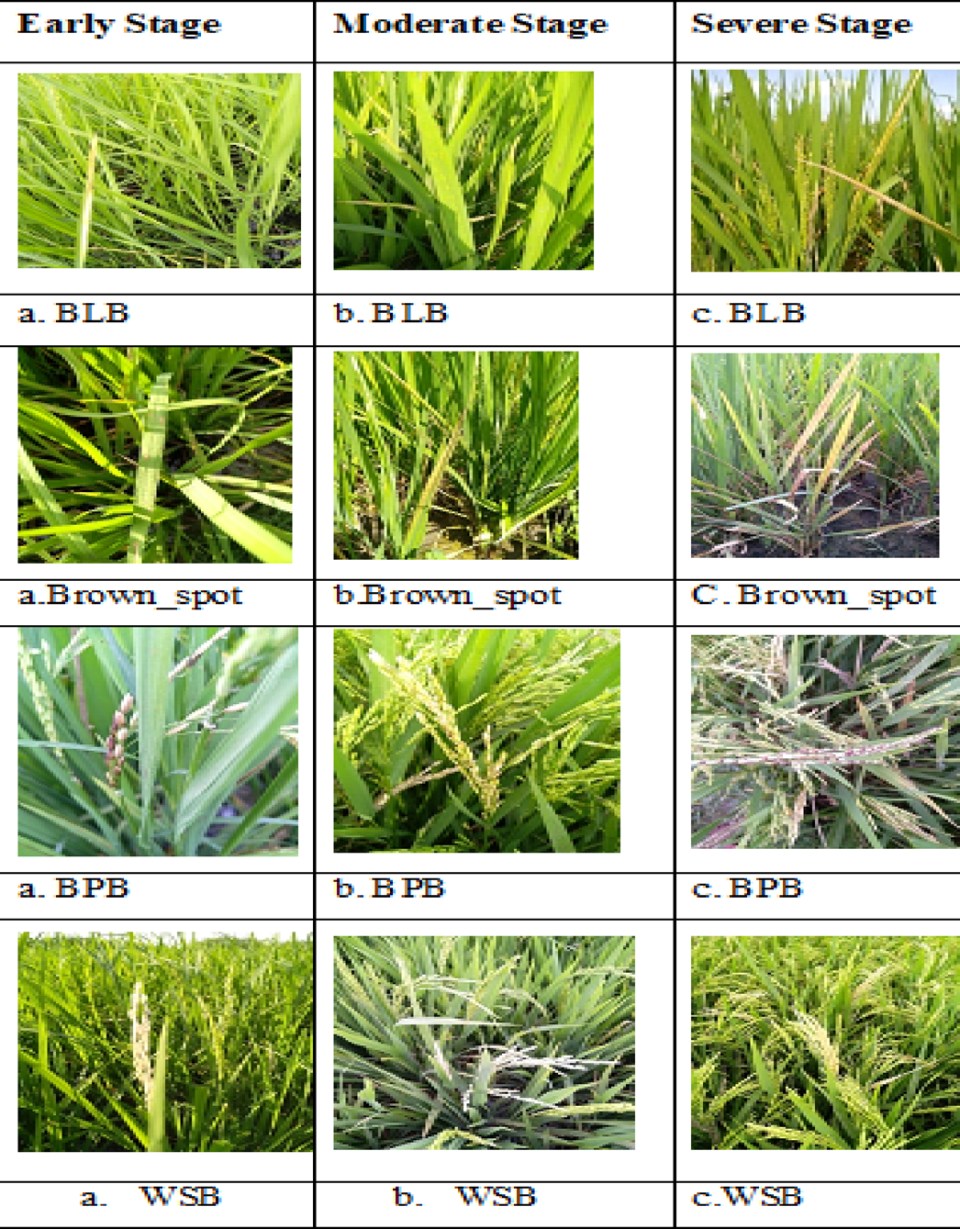
Progression of paddy disease in three stages.

There are three phases to the disease’s progression (40–80 days). Stage 1 (Early): Minor symptoms, such as tiny patches or yellowing. Stage 2 (Moderate): spreading infection, leaf bending, and larger lesions. Stage 3 (Severe): Significant wilting, necrosis, and damage that reduces yield. Control depends on early detection. The disease courses of brown spot, white stem borer, bacterial leaf blight, and bacterial panicle blight are depicted in Fig. 8. Bacterial leaf blight (a) and white stem borer (b, c) go from minor leaf discoloration to complete leaf destruction.

From minor lesions to significant leaf loss and plant stress, Brown Spot (Row 2) develops. Grain sterility and total panicle drying are the severe symptoms of Bacterial Panicle Blight (Row 3), which begins with minor discoloration and progresses to a major illness. Beginning with modest white spikes, White Stem Borer (Row 4) causes damage to the larvae and, in more severe cases, slowed growth, yield loss, and large white spikes.

Table 4 examines the parameters, complexity, strengths, and shortcomings of six deep learning models: Inception-ResNet v2, VGG19, ResNet101, Inception v3, Xception, and SSDHR Classifier. Despite its speed, Inception-ResNet v2 (164 layers) is prone to overfitting. Although VGG19 (19 layers) has a significant computational cost, it obtains good accuracy. Although ResNet101 (101 layers) is resource-intensive (163.2 MB), it is generalisable. Though it runs the danger of overfitting, Inception v3 (48 layers) effectively retrieves characteristics. With depth-wise separable convolutions, Xception (312 layers) improves efficiency but maintains complexity. Although SSDHR (18 layers) is precise and lightweight, its feature extraction depth is constrained. The table illustrates the trade-offs between generalisability, depth, accuracy, and efficiency.

**Table 4:** Comparison of deep learning models on their parameters, complexity, strengths, and weaknesses.

Model	Parameters (MB)		Complexity	Strengths	Weaknesses
	Total	Trainable			
Inception—ResNet v2	0.29	3.01	Complex (164 layers)	Simple and fast	Low accuracy, overfitting risk
Vgg19	77.4	1.01	Very Deep (19 layers)	High accuracy	Very deep, computationally expensive
ResNet101	163.2	513.27	Deep (101 layers)	High generalizability	Requires training time
Inception v3	87.18	4.01	Complex (48 layers)	Efficient feature Extraction	Overfitting risk
Xception	80.08	513.27	Complex (312 layers)	depth-wise separable convolutions	Computational complexity
Inception-ResNet v2	0.29	3.01	Complex (164 layers)	Simple and fast	Low accuracy, overfitting risk
SSDHR classifier	11.62	11.61	Highly customizable,	High accuracy	Less computational complexity

Plant disease detection with different machine learning models is the main topic of Table 5. According to Turkoglu et al., LSTM performed better than SVM in detecting apple illness (98.20%). With an accuracy of 95.31%, Patil & Kumar’s CNN-LSTM hybrid (Paddy-Fusion) outperformed unimodal models. Severity was successfully categorized by CNN-LSTM models developed by Lamba et al. (92%) and Kukreja et al. (94.06%). CNN-LSTM’s effectiveness in treating paddy illnesses was validated by Kaur et al. (94.84%). The accuracy of Choubey & Dubey’s CNN + ABi-LSTM with feature selection was 98.86%. Jiang et al. showed that CNN was more than 95% successful in identifying paddy illnesses. Outperforming all models, the suggested SSDHR classifier had the greatest accuracy (99.25%).

**Table 5:** Comparison of accuracy with existing models.

S. No	Author(s)	Methodology	Key findings	Accuracy (%)
1	Turkoglu et al.^13^	Pre-trained models + LSTM and SVM classifiers	The LSTM classifier outperformed SVM in accuracy in apple disease detection	98.20
2	Patil & Kumar et al.^14^	CNN-LSTM hybrid (Paddy-Fusion)	Outperformed unimodal approaches (e.g., CNN, MLP)	95.31
3	Lamba et al.^15^	CNN-LSTM	Achieved varied accuracies for severity classes	92
4	Kukreja et al.^18^	LSTM-CNN hybrid model using temporal and spatial data	Effective for determining disease severity	94.06
5	Kaur et al.^16^	CNN-LSTM hybrid for Paddy Sheath Rot Disease	Accurate severity classification using temporal and spatial data	94.84
6	Choubey & Dubey^19^	CNN + ABi-LSTM with SVM-RFE + ARO for feature selection	High accuracy in plant leaf disease detection	98.86
7	Jiang et al.^17^	CNN for leaf image classification	High accuracy in detecting bacterial leaf blight, brown spot, and paddy blast	> 95
8	Proposed model	SSDHR classifier	High accuracy	99.25

The comparative analysis presented in the Table 6 highlights the performance of various state-of-the-art models for paddy disease classification. Among the evaluated models, the proposed model achieved the highest accuracy of 99.25%, along with precision, recall, and F1-score all at 99.2%, demonstrating its robustness and reliability in early disease detection. Models such as CNN + ABi-LSTM + SVM-RFE + ARO and Inception-ResNet v2 also exhibited strong performance, with accuracies of 98.86% and 98.2% respectively. Traditional CNN and LSTM hybrid models like those proposed by Patil & Kumar and Kukreja et al. showed relatively lower accuracies of 95.31% and 94.06%, respectively. Other deep learning architectures such as Xception and ResNet101 performed well, with accuracies of 97.2% and 97.7%, but still lagged behind the proposed framework. The consistent outperforming of the proposed model across all metrics indicates its superior ability to extract and utilize both spatial and temporal features for more accurate and early disease identification, making it highly suitable for real-world agricultural applications.

**Table 6:** Performance of various state-of-the-art models for paddy disease classification.

S. No	Model	Accuracy (%)	Precision (%)	Recall (%)	F1-Score (%)
1	CNN + ABi-LSTM + SVM-RFE + ARO	98.86	98.8	98.8	98.8
2	Inception—ResNet v2 (Turkoglu)	98.2	98.2	98.2	98.2
3	CNN for Leaf Image (Jiang et al.)	> 95	~ 96.0	~ 96.0	~ 96.0
4	CNN-LSTM Hybrid (Patil & Kumar)	95.31	95.3	95.3	95.3
5	LSTM-CNN Hybrid (Kukreja et al.)	94.06	94.1	94.1	94.1
6	CNN-LSTM (Lamba et al.)	92	92	92	92
7	Inception v3	96.8	95.8	95.65	95.7
8	Xception	97.2	96.61	96.58	96.57
9	ResNet101	97.7	97.52	97.5	97.5
10	VGG19	93.66	93.49	93.19	93.2
11	Proposed model	99.25	**99.2**	**99.2**	**99.2**

Significant values are in [bold]

## Conclusion and future scope

In this work, we introduced a novel deep learning framework that integrates Self-Supervised Deep Hierarchical Reconstruction (SSDHR) and Long Short-Term Memory (LSTM) networks for the accurate and early detection of paddy crop diseases. Unlike traditional methods that rely solely on spatial features, our model leverages both spatial and temporal cues, enabling continuous monitoring and early recognition of subtle changes in crop health. The use of multi-scale convolutional branches within SSDHR enhances the extraction of diverse discriminative features, while the Symmetric Fusion Attention mechanism improves feature selection by fusing spatial and temporal information effectively. Furthermore, the XGBoost classifier contributes to stable and robust classification outcomes. Experimental results validate the effectiveness of our model, achieving a high classification accuracy of 99.25% across 13 different paddy disease categories. The proposed approach demonstrates significant potential for deployment in real-world agricultural monitoring systems. In the future scope localization will be integrated with time-series databases, to overcome the problems of regional and temporal variability in agriculture.

## Data Availability

Yes, I have research data to declare. “The data supporting this study are available in the IEEE Data Port.” Dataset Link: https://ieee-dataport.org/documents/paddy-doctor-visual-image-dataset-automated-paddy-disease-classification-and-benchmarking
